# Factors Associated with Human Brucellosis among patients Attending in Ayu Primary Hospital, North Showa, Ethiopia: ACase Control Study

**DOI:** 10.4314/ejhs.v31i4.4

**Published:** 2021-07

**Authors:** Bahiru TeshomeYimer, Berhanu Elfu Feleke, Kassawmar Angaw Bogale, Gebiyaw Wudie Tsegaye

**Affiliations:** 1 Debreberhan city administration health office, North Showa Zone, Debreberhan, Ethiopia; 2 Department of Epidemiology and Biostatistics College of Medicine Health Sciences, Bahirdar University, Ethiopia

**Keywords:** Human Brucellosis, determinant, zoonosis, Ethiopia

## Abstract

**Background:**

Brucellosis is a disease of domestic and wild animals commonly caused by Brucella species and can be transmitted to humans (zoonosis). Susceptibility to Brucellosis in Humans depends on immune status, routes of infection, size of the inoculums, and to some extent, the species of Brucella. Globally more than 500,000 new cases are reported each year. In sub-Saharan Africa, Brucellosis prevalence is unclear and poorly understood with varying reports from country to country, geographical regions as well as animal factors.

**Methods:**

Facility-based unmatched case-control study was conducted on 167 patients with human brucellosis and 332 controls from February 27/2019 to May 20/2019 in AYU primary hospital, North Showa Zone, Ethiopia. descriptive statistics such as frequency and percentages were used to describe the profile of case and control and analytical statistics such as bivariate and multivariate logistic regression analysis was performed to identify the determinants of human brucellosis.

**Result:**

A total of 499 participants were included with a response rate of 99.60%. The mean age of participants was 45.46 years with a standard deviation (SD) of ±12.96 years. Human brucellosis had a statistically significant association with raw milk consumptions (AOR 5.75[95%CI 1.97–16.76]), slaughtering of animals at home(AOR 14.81[95%CI 3.63–60.38]), having contact with animal manure(AOR 2.87 [CI 1.08–7.62]), having contact with aborted cattle's fetus (AOR 3.01[95%CI 1.34–9.13]) and knowledge about brucellosis(AOR 0.29 [95%CI 0.08–0.83].

**Conclusion:**

Generally in this study knowledge about Human Brucellosis, contact with animal manures, practicing animal slaughtering at home, having contact with animal ruminants, and consuming raw milk were identified as determinants for human brucellosis infection.

## Introduction

Brucellosis is a disease of domestic and wild animals commonly caused by Brucella species and can be transmitted from animals to humans (zoonosis) (1). There are six Brucella species; four of which are zoonotic. The zoonotic species in order of decreasing virulence in humans are Brucella melitensis, Brucella suis, Brucella abortus, and Brucella canis (2,3). Susceptibility to Brucellosis in humans depends on various factors, including the immune status, routes of infection, size of the inoculums, and, to some extent, the species of Brucella (2). In general, B. melitensis and B. suis are more virulent for humans than B. abortus and B. canis, although serious complications can occur with any species of Brucella (3,4).

The most common clinical features of Brucellosis in humans include fever, fatigue, headache, sweating, loss of appetite, muscular pain, lumbar pain, and weight loss (4). complications may include Arthritis, sacroiliitis, spondylitis, and disorders of the central nervous system(3). Brucella can cause abortions in women mostly in the first and second trimesters of pregnancy while males can exhibit epididymo-orchitis (5). the disease primarily presents as a fever of unknown origin with multiple clinical signs and symptoms(5). Patients regularly suffer serious focal complications such as spondylitis, neurobrucellosis, or Brucella endocarditis (6). the clinical features and presentation of human Brucellosis overlap with many other infectious and non-infectious diseases such as typhoid fever, rheumatic fever, spinal tuberculosis, and tumors (7–9). as the clinical picture is not specific laboratory testing should support the diagnosis(8,9).

Risk factors influencing the occurrence of Human Brucellosis are socio-demographic factors, mode of transmission, contact with animals and animal products, participant's involvement in milking, sharing water sources with animals, assisting animals to give birth or drink animal urine(4, 8, 10). Prevention of the disease includes education to avoid consuming unpasteurized milk and milk derivatives, barrier precautions for hunters and professionals at risk, careful handling and disposal of afterbirths (6, 11) The combination of positive Rose Bengal Plate test(RBPT) and serum agglutination test (SAT) is a good diagnostic criterion with 80% specificity and 100% sensitivity among serological tests (10, 11).

According to the World Health Organization (WHO), worldwide more than 500,000 new cases of brucellosis are reported each year (4,5). The reported incidence in brucellosis endemic areas varies widely from <0.01 to >200 per 100,000 population(5,10). In sub-Saharan Africa, brucellosis prevalence is unclear and poorly understood with varying reports from country to country, geographical regions as well as animal factors (11, 14), for example among the African countries Algeria is the leading country with brucellosis in human worldwide(4, 13). The burden of human brucellosis is also higher among other Sub-Saharan and East African countries. Studies from central Uganda revealed 17% prevalence of human brucellosis among agro-pastoral communities (15). Other pieces of evidence from Togo and Libya also revealed even much more prevalence of human brucellosis 41% and 40% respectively (16,17). a meta-analysis done in Ethiopia reported that the seroprevalence of human brucellosis was 6.7% (18). similarly, an institutional based study done in Jimma hospital, Ethiopia showed a seroprevalence of 3.6% (19).

Studies conducted both in developing as well as developed countries including Ethiopia were mainly on the prevalence(burden) of brucellosis and focused on animals related studies(18). Human brucellosis in Ethiopia appears to have been under-diagnosed (18). and there is limited evidence on the determinants of human brucellosis in the study area despite there is occasional episodes of the outbreak. Therefore; identifying determinants of human brucellosis using advanced study design is quietly valuable. So this study was done to identify the determinants of human brucellosis in North Showa Zone, Ethiopia.

## Methods

Un-matched case-control study was conducted from February 27/2019 to May 20/2019 in Ayu Primary Hospital, North Showa Zone, Amhara Region, Ethiopia. According to the 2018 report, the zone has a population of 2,226,698. The zone has 10 Hospitals, 95 Health centres, 430 Health posts, and 102 private health institutions. Brucella diagnosis and treatment is given only in Ayu primary Hospital. The hospital has more than 112 health professionals and provide services like Laboratory, Orthopaedics, Ophthalmic, Surgical, Obstetrics, Gynaecological, Pharmaceutical, and Dental services. All patients attended Ayu Hospital during the period were taken as study population. Those patients having clinical feature of Brucellosis and positive test results for both Rose Bengal Plate Test (RBPT) and Serum Agglutination Test (SAT) were study subjects, while relatives or neighbours attended the same hospital of the case, but free of the clinical feature of brucellosis and have negative results for SAT were the study population for controls. Double population proportion formula was used to determine the sample size using Epi-Info version7. Patients who have positive results only for one test (RBPT/ SAT), those with clinical feature of brucellosis and other febrile illness and those who were not able to communicate were excluded.

Human Brucellosis (yes, no) was the dependent variable while, the independent variables were socio-demographic variable (sex, age, residence education status, occupation), behavioural and environmental factors (raw milk consumption, eating raw meat, drinking uncooked blood, consuming the product of raw milk, information about Brucellosis) and environmental factors (assisting cattle delivery, family size, milking, assisting during cattle abortion, contact with placenta, contact with manure, infected household member, home slaughtering, cleaning of animal house and body).

The animal-related occupation was taken as the main research hypothesis variable (6, 14, 22) and the assumptions made for the sample size calculation were 95% confidence interval, 5% marginal error, 80% power, a ratio of control to case 2:1, the final sample size was estimated to be 501(167 cases and 334 controls) assuming 10% non-response rate. The 167 cases were selected by using systematic sampling technique and the previous average two months report was used to determine the constant interval each case were selected in every 2 intervals from those who presented with the clinical feature of human brucellosis (fever, fatigue, joint pain, sweating, chills, headache) and positive laboratory test while controls (334 participants) were selected from the same area where the cases came from (neighbours or relatives of cases that were coming with the case as supporters or caregivers).

Data on general socio-demographic, behavioural and environmental characteristics were collected using a pre-tested structured interviewer administered questionnaire while the disease status of the participants was (human brucellosis infection) was determined using laboratory tests and clinical features. approximately 5 ml of blood was collected from each patient in evacuated plain vacutainer tubes. The Rose Bengal plate test (RBT) antigen method prescribed by the center for disease control was used. The test was undertaken at Ayu Primary Hospital laboratory. 30 µl serums were mixed with an equal volume of antigen on a white tile or enamel plate to produce a zone approximately 2 cm in diameter. The antigen and serum were mixed thoroughly using an applicator stick (a stick being used only once) and the plate was rocked by a shaker for about 4 minutes. Then, the mixture was examined for agglutination in a good light. According to the degree of agglutination, the result was visually graded on a scale from 0 to 3 as follows: 0 = no agglutination, + = barely perceptible, ++ = fine agglutination, some clearing, +++ = Coarse clumping, definite clearing. Those samples identified with no agglutination were recorded as negative whereas, those with +, and above were recorded as positive. all the RBT positive samples were re-tested by serum agglutination test with the dilution>=1:160(12). To assure the quality of data, Data collectors and supervisor were trained on data collection procedures and the questionnaire was first prepared in English then translated in Amharic [local language] then back-translated into English to keep its consistency. At the end of each interview, the supervisor had cross-checked the questionnaire to ensure completeness and data accuracy. Data were entered into Epi info version 7 statistical software and exported to SPSS statistical software. Descriptive analysis was done on the frequency distribution of selected sociodemographic characteristics. Simple binary logistic regression was done to identify factors associated with human brucellosis, variables that had a p-value of less than0.2 in the simple binary logistic regression analysis were included in multivariate analysis. The model fitness was tested by Hosmer and Lemeshow goodness test. The strength of association between the dependent variable and independent variable was expressed by odd ratio, 95% Confidence interval, and p-value. Variables with a p-value less than 0.05 in multivariate analyses were considered significant.

Ethical clearance was obtained from the institutional review board (IRB) of Bahir Dar University, College of Medicine and Health Science. Permission letter was obtained from North Showa Zonal Health Department to Ayu Primary Hospital. Informed consent was obtained from each respondent. Those participants having positive results were link to their physician and got appropriate treatment and health education. Data collectors were trained in infection prevention to prevent infection from the patient as well as from the data collector to the participants.

## Results

A total of 499 participants were included. Among these 167 were cases of Human Brucellosis and the remaining 332 were controls. From the total participants, 92 (18.4%) were females and 407 (81.6%) were males. the mean age of the participants was 45.5 years with a standard deviation (SD) of± 12.9 years.

**Profile of cases and controls**: One hundred sixty-seven patients who had Brucellosis were included in this study making the response rate100% among cases. The mean age of cases was 43.6years (SD±12.99) and 74.8% of cases were males. For controls, a total of 332 clients who had no Human Brucellosis were included with a response rate of 99.4%. The mean age of the controls was 46.4years (SD12.85) and 84.9% of controls were males (Table 1).

**Table 1:** Profile of cases and controls in Ayu Hospital, North Showa, Ethiopia (N=499)

Profile of cases				Profile of controls			

Variables	Categories	Frequency	%	Variables	Categories	Frequency	%
Age	≤ 44	90	53.8	**Age**	≤ 44	142	42.77
	≥44	77	46.2		≥44	190	57.23
Occupation	Student	1	0.6	**Occupation**	Student	5	1.50
	Housewife	1	0.6		Housewife	10	3.03
	Employers	2	1.2		Employers	12	3.61
	Farmers	42	25.2		Farmers	160	48.19
	Animal related	121	72.5		Animal related	145	43.67
Sex	Male	42	25.2	**Sex**	Male	282	84.9
	Female	125	74.8		Female	50	15.0
Educational status	Illiterate	132	79.1	**Educational** **status**	Illiterate	236	71.08
	Primary	0	0.0		Primary	6	1.80
	Secondary and above	35	20.9		Secondary and above	90	27.12
Residence	Afar	34	20.4	**Residence**	Afar	30	9.04
	Amhara	125	74.9		Amhara	287	84.44
	Oromo	8	4.8		Oromo	15	6.52
Marital status	Single	14	8.4	**Marital** **Status**	Single	39	11.75
	Married	140	83.8		Married	269	81.02
	Divorced	12	7.2		Divorced	21	6.32
	Widow	1	0.6		Widowed	3	0.91

**Determinants of human brucellosis**: Simple binary logistic regression was done initially to identify candidate variables. Variables with a p-value of less than 0.25 were selected as a candidate and entered into a multivariable binary logistic regression to determine the association between different independent variables with Human Brucellosis. Among all independent variables sex, educational status, ethnicity, age, raw milk consumption, raw meat consumption, raw milk product consumption, not washing knives and hands after contact with meat, slaughtering animal at home, consumption of uncooked blood, contact with animal manure, contact with aborted foetus and placenta, keep weak new-borns at home, fetching water from an unsafe source, sharing the same water source with animals, and knowledge about Human Brucellosis were adjusted. Among these determinants raw milk consumptions, slaughtering of animals at home, having contact with animal manure, having contact with an aborted foetus, and knowledge about Brucellosis had a statistically significant association with Human Brucellosis infection. individuals who slaughter animals at home were fifteen times more likely to develop Human Brucellosis compared to individuals who did not slaughter animals at home (AOR 14.8[95%CI 3.6–60.4]). People who consume raw milk were six times more likely to develop Human Brucellosis than those who did not consume raw milk (AOR 5.7[95%CI 1.9–16.7]). Individuals who had good knowledge about Human Brucellosis were 72% less likely to develop Human Brucellosis compared to those who had poor knowledge about Human brucellosis (AOR 0.28[95%CI 0.08–0.82]). People who had contact with an aborted foetus of animals were three times more likely to develop Human Brucellosis than those who had no contact (AOR 3.01[95%CI 1.34–9.13]). those individuals who had contact with animal manures were two times more likely to develop Human Brucellosis compared to those who had no contact with animal manures (AOR 2.87 [CI 1.08–7.62]) (Table 2).

**Table 2:** Bi-variety and multivariate analysis on determinants of Human Brucellosis in Ayu Hospital, North Showa Zone Ethiopia, 2019 (N=499)

Variable case	Category	COR(95%CI)		AOR(95%CI)	P-value
			
	Case	Control			
Educational status					
Illiterate	33	75	1.53(1.01–2.38)	1.23(0.39–3.87)	0.71
Literate	134	157	1	1	
Ethnicity					
Oromo	8	15	0.467(0.29–0.74)	0.49(0.16–1.57)	0.23
Amhara	125	287	0.38 (0.23–0.91)	0.41(0.21–1.34)	0.21
Afar	34	30	1	1	
Consume fresh milk					
Yes	126	95	7.66(5.01–11.73)	5.75(1.97–16.76)	0.001
No	41	237	1	1	
Boil milk					
Yes	129	144	4.43(2.91–6.76)	1.48(0.39–5.57)	0.56
No	38	188	1	1	
Consume raw milk product					
Yes	40	39	2.36(1.45–3.85)	0.61(0.14–2.72)	0.52
No	127	293	1	1	

Eat raw meat					
Yes	115	38	4.28(2.85–6.42)	2.45(0.9–6.66)	0.08
No	52	294	1	1	
Wash your hand					
Yes	100	291	0.21(0.13–0.33)	0.55(0.20–1.52)	0.25
No	67	41	1	1	
Wash knife					
Yes	102	279	0.29(0.19–0.46)	0.77(0.08–7.62)	0.82
No	65	53	1	1	
Slaughter at home					
Yes	141	95	13.53 (8.36–21.8)	14.81(3.63–60.38)	<0.01
No	26	237	1	1	
Clean animal body/structure					
Yes	141	162	5.69(3.53–9.11)	1.76(0.47–6.61)	0.40
No	26	170	1	1	
Consume blood					
Yes	115	42	16.14(10.14–25.7)	1.92(0.48–7.56)	0.35
No	52	292	1	1	
Contact with animal manure					
Yes	67	66	2.70(1.79–4.07)	2.87(1.08–7.62)	0.03
No	100	266	1	1	
Help during delivery					
Yes	78	58	4.14(2.73–6.27)	0.60(0.18–1.95)	0.39
No	89	274	1	1	
Contact with placenta and abortion					
Yes	98	59	6.57(4.33–9.97)	3.01(1.34–9.13)	0.04
No	69	273	1	1	
Keep weak new baby animal at home					
Yes	132	188	2.89(1.88–4.45)	0.32(0.08–1.22)	0.09
No	35	144	1	1	
Water source					
Safe	135	228	1.92(1.23–3.02)	2.41(0.84–6.91)	0.10
Unsafe	32	104	1	1	
Sex					
Male	125	282	1.89(1.19–3.01)	0.82(0.25–2.73)	0.75
Female	42	50	1	1	
Age					
≤ 44	90	142	0.64(0.44–0.93)	1.19(0.39–3.63)	0.75
≥ 44	77	190	1	1	
Knowledge about brucellosis					
Good	11	68	0.07(0.03–0.15)	0.28(0.08–0.83)	0.02
Poor	61	26	1	1	

## Discussion

A case-control study was conducted on 499 participants (167 patents with Human Brucellosis and 332 clients with no Human Brucellosis) to identify the determinants of Human Brucellosis. Human Brucellosis was significantly associated with animal slaughtering area. Individuals who slaughter animals at home were fourteen times more likely to develop Human Brucellosis compared to individuals who did not slaughter animals at home (AOR 14.8[95%CI 3.6–60.4]). this finding was in line with studies done in Tunisia, Iran, Kenya, and Tanzania (19,25, 26,33). These might be during slaughtering animals at home individuals may expose and have increased risk of contact with blood, ruminates, manures, animal bodies, and others which will increase the risk of developing human Brucellosis.

People who consume raw milk were almost 6 times more likely to develop Human Brucellosis than those who did not consume raw milk (AOR 5.7[95%CI 1.9–16.7]) which is similar to findings reported from Brazil and Tanzania (9, 15, and 22). Similar studies conducted in Sudan, Cameroon, and Egypt also reported a higher risk of Human Brucellosis due to consumption of raw milk (27, 28, and 29). These might be since Brucella needs PH from slightly acidic to neutral media which was similar to the PH of fresh milk so that consuming the raw milk may favour the growth of the bacteria and facilitate the transmission of the disease. Individuals who had good knowledge about Human Brucellosis had a 72% lower risk of developing human Brucellosis as compared to those who had poor knowledge about the disease (AOR 0.28 [95%CI 0.079–0.826]) this finding is consistent with studies reported from Iran and Cameron (5, 28). The reason might be that people having knowledge about the disease will interrupt the mode of transmission, minimizing contact with animals and its product and have restricted consumption of raw animal products and might wash their hands and other materials after having contact with animals.

Individuals who had contact with an aborted foetus of animals were three times more likely to develop Human Brucellosis compared to those who had no contact (AOR 3.01[95%CI 1.34–9.13]). This finding was similar to the studies done in Georgia, Tanzania Iran, and Cameron (14, 19, 25, and 28). in a rural part of Ethiopia, livestock delivery is often assisted with bare hands, and consuming raw milk is a common practice in a significant segment of the population. Given that Brucella spp. are known to have a predilection for reproductive organs particularly placenta and aborted foetuses, it is logical that assisting animals in delivery increases the risk of infection (26). this is supported by the fact that assisting animals during abortion and handling of the parturient product increases the risk of developing Brucellosis and facilitate its transmission.

Contact with animal manures was associated with an increased risk of developing Human Brucellosis; those individuals who had contact with animal manures were almost three times more likely to develop Human Brucellosis compared to those who had no contact with animal manures (AOR 2.87 [CI 1.08–7.62]) which is consistent with findings from a similar study conducted in Georgia (23) this might be due to the reason that during contact with the manure the individuals might have a high risk of exposure to the bacteria.

Participants were in the age group between 18 and 84 years, and cannot represent the entire population. Another limitation is related to the selection of the participants, which excluded the possibility of inviting other residents that have not sought health services during the period.

In this study poor knowledge about Human Brucellosis, contact with animal manures, practicing animal slaughtering at home, having contact with animal abortus ruminants, and consuming raw milk were significantly associated with Human Brucellosis infection. Therefore, health education about the mode of transmission of Human Brucellosis and awareness creations about the disease to the community by a health professional should be done regularly, health sectors should focus on educating the population about the risk of consuming raw milk, and animal and health sector should coordinate and educate the community for slaughtering animals in a separate animal slaughtering house.
